# Heterogeneity of differential atmosphere perception and its relationship with organizational silence among Chinese nurses: a cross-sectional study using latent profile analysis

**DOI:** 10.3389/fpsyg.2025.1592094

**Published:** 2025-05-27

**Authors:** Qiaoli Bai, Yaping Bai

**Affiliations:** Department of Burns and Cutaneous Surgery, Xijing Hospital, Air Force Medical University, Xi’an, China

**Keywords:** clinical nurse, differential atmosphere perception, organizational silence, latent profile analysis, China

## Abstract

**Objective:**

To probe the heterogeneity and the influences of clinical nurses’ perceptions of differential atmosphere, and to investigate the relationship between each profile of differential atmosphere perception and organizational silence.

**Methods:**

We adopted the General Information Questionnaire, the Differential Atmosphere Scale, and the Nurses’ Organizational Silence Assessment Questionnaire to survey 523 clinical nurses in three tertiary general hospitals in Shaanxi Province. And we estimated the potential categories of clinical nurses’ differential atmosphere perceptions via latent profile analysis, and quantified the influences on each profile via χ^2^ and logistic regression analyses, and probed the differences in the organizational silence scores of individuals with three differential atmosphere perception profiles through the least significant difference (LSD) method.

**Results:**

The differential atmosphere perception of clinical nurses was divided into “Insiders” (16.25%), “Go-betweens” (57.55%), and “Outsiders” (26.20%). Monthly income and years of working experience were the influencing factors of different categories of nurses’ differential atmosphere perceptions. There was a significant difference on the total organizational silence score and on the three profiles (*p* < 0.001), specifically, “Outsiders” > “Go-betweens” > “Insiders.”

**Conclusion:**

This study has identified three distinct subgroups in the differential atmosphere perception of nurses and their influencing factors. The findings demonstrated the heterogeneity among the clinical nursing population. Nursing managers may take prompt and targeted measures to reduce differential atmosphere perception, so as to improve the development of the nursing team.

## Introduction

Globally, nurses serve a crucial role in advancing universal health care, achieving the sustainable development goals, and promoting population health outcomes (Teutsch et al., 2025). The world is currently facing the dual challenges of a severe shortage of nursing human resources and the increasing demand for nursing services (Chen et al., 2024). In the pursuit of high-quality nursing development, organizational management factors cannot be overlooked.

The differential sequence pattern is a socio-psychological concept rooted in the traditional cultural characteristics of Chinese society (Qu et al., 2024). When introduced into the field of organizational management, it gave rise to research on the perceived climate of difference. Differential atmosphere perception refers to an individual’s perception of the degree of relational distance from leadership and the inequitable distribution of resources (Pan et al., 2023; Zhang et al., 2021). This perception is reflected in two main areas: the perception of “difference,” such as differences in treatment and unequal distribution of resources; and the perception of “order,” such as hierarchies of authority and relationship-orientation (Zhang et al., 2021). Studies have shown that a higher level of differential atmosphere perception not only reduces team cohesion and nurses’ job satisfaction but also triggers negative behaviors such as organizational silence, which diminishes work efficiency and may even lead to turnover intentions, significantly undermining the stability of the nursing workforce (Zhang et al., 2021).

Organizational silence refers to the behavior of nurses withholding their opinions or perspectives on existing or potential issues in the workplace due to various considerations (Zhang et al., 2021). The formation of a silent atmosphere hinders communication efficiency among healthcare professionals, impairing information exchange and jeopardizing the harmony, stability, and long-term development of departments (Alhojairi et al., 2024). According to social exchange theory (El-Gazar and Zoromba, 2024; He et al., 2022), the interaction between nurses and departments is essentially a reciprocal exchange of benefits, and the internal differential treatment and management climate within an organization can influence nurses’ cognitive attitudes and behavioral patterns.

Previous research has paid limited attention to clinical nurses’ differential atmosphere perception, often adopting variable-centered approaches to explore its influencing factors and its relationship with organizational silence (Zhang et al., 2021), while neglecting the heterogeneity within the clinical nurse population. Latent profile analysis, a person-centered research method, can more accurately identify the heterogeneity in clinical nurses’ differential atmosphere perception based on response patterns. Therefore, this study aimed to clarify the heterogeneity and influencing factors of clinical nurses’ differential atmosphere perception and to explore the differences in organizational silence among nurses with varying levels of differential atmosphere perception, aiming to provide insights for nursing managers to implement targeted incentive strategies.

## Aims and hypotheses

This study aimed to identify the various subgroups of differential atmosphere perception among Chinese nurses using latent profile analysis, examine the demographic factors associated with differential atmosphere perception profiles, and then explore the relationship between latent categories and organizational silence. The study is structured on the following three hypotheses:

*Hypothesis 1*. There are subgroup differences in differential atmosphere perception among nurses.

*Hypothesis 2*. Nurses’ differential atmosphere perception profiles vary across demographic and work-related characteristics.

*Hypothesis 3*. Membership in various differential atmosphere perception profiles can influence nurses’ organizational silence.

## Methods

### Design

A cross-sectional design was conducted from July to August 2023. This study followed the STROBE (strengthening the reporting of observational studies in epidemiology) statement.

### Participants

This study was a cross-sectional survey conducted from July to August 2023 in 3 tertiary hospitals (from Xi’an, Xianyang, and Yulin) in Shaanxi Province, China. All eligible nurses were invited to participate in this study if (1) they were the registered nurse and have passed the National Nursing Licensure Examination (NNLE); (3) they worked full-time with at least 1 year of clinical nursing experience; (3) they voluntarily participated in this study. Nurses who were not on duty due to vacation or illness were excluded. A total of 561 nurses participated in this study. After excluding 38 invalid questionnaires (e.g., those with identical answers for all items or unrealistic responses), 523 nurses were ultimately included in the final analysis.

The majority of the respondents were female (*n* = 506, 96.7%), and their median age was 25 years (IQR 26–33) (see Table 1).

**Table 1:** Characteristics of the participants (*n* = 523).

Variable	Category	*N* (%)
Gender	Male	17 (3.3)
	Female	506 (96.7)
Age (years) (R: 22–55)	20–30	301 (57.6)
	31–40	189 (36.1)
	>40	33 (6.3)
Education	College degree	169 (32.2)
	Bachelor’s degree or above	354 (67.7)
Title	Primary nurse	153 (29.3)
	Senior nurse	246 (47.0)
	Nurse-in-charge and above	124 (23.7)
Years of nursing work	1–5 years	215 (41.1)
	6–10 years	177 (33.8)
	>10 years	131 (25.1)
Department	Internal medicine	299 (57.2)
	Surgery	88 (16.8)
	ICU/operating room/emergency department	73 (14.0)
	Others	63 (12.0)
Days of night shift monthly	0	58 (11.1)
	1–7	160 (30.6)
	>7	305 (58.3)
Monthly income (CNY)	<5,000	100 (19.1)
	5,001–10,000	173 (33.1)
	10,001–15,000	180 (34.4)
	>15,000	70 (13.4)

N (%): number and percentage; R: range; ICU: Intensive care unit; CNY: Chinese Yuan.

### Sample size

Previous studies have confirmed that hybrid models such as latent profile analysis and latent category analysis with sample sizes of 500 and above are more suitable for data analysis. The results are also more reliable (Chen et al., 2024; Jiang et al., 2023).

### Measurement

#### General questionnaire

The general demographic characteristics questionnaire was designed by the research team. Demographic characteristics included gender, age, and education. Work-related characteristics included professional title, years of nursing work [1–5 years (initial stage of nurse career development), 6–10 years (proficient stage), >10 years (senior specialist stage)] according to practical experience in clinical management and the concept of nurse career development in China, department, days of night shift monthly, and salary.

#### Differential atmosphere perception scale

The Chinese version of Differential Atmosphere Perception Scale was developed by Liu (2003) and verified by Liu et al. (2009). The scale consists of 11 self-reported items in 3 dimensions: favoritism (3 items), interdependence (6 items), and trusted roles (2 items). Responses were rated on 5-point Likert scale (1 = strongly disagree; 5 = strongly agree), with a total score of 11–55. The higher the score, the higher the individual’s perception of different atmospheres. In the literature, a Confirmatory Factor Analysis was conducted on the scale with its 11-item and three-factors, showing good data fit (χ^2^/df = 2.139, CFI = 0.952, TLI = 0.926, SRMR = 0.043, RMSEA = 0.068) (Zhu and Xie, 2018). In this study, a Cronbach’s alpha of 0.858 was found in this sample and is considered satisfactory.

#### Nurse organizational silence assessment questionnaire

The questionnaire was developed by Yang and Yang (2016) and consists of 20 items and 4 dimensions: negative silence (6 items), defensive silence (6 items), prosocial silence (4 items), and indifferent silence (4 items). The questionnaire employs a Likert 5-point scale, with scores ranging from 1 to 5, representing “never felt” to “always felt,” respectively. The total score ranges from 20 to 100, with higher scores indicating more pronounced silent behavior in the workplace. The Cronbach’s alpha coefficient for the original scale amounted to 0.918 and was 0.874 in this study.

#### Data collection

Prior to conducting this study, we sought the support and assistance of the nursing department director and ward nurse managers. The QR codes for the electronic questionnaire were distributed by the nursing head to the work chat group to facilitate data collection. The survey homepage included clear and standardized instructions, explaining the purpose and content of the study, as well as assuring participants of data confidentiality. Clinical nurses provided informed consent and were free to withdraw from the survey at any time. The questionnaire platform was configured to require responses for all items and ensured anonymous submission. Additionally, each IP address was restricted to a single submission to prevent duplicate responses. Two researchers rigorously check the data relating to demographic and working characteristics to identify potential duplicate responses from participants with identical profiles. Submission timestamps, response durations, and IP locations were additionally analyzed to eliminate duplicate entries. We also excluded those with illogical responses, obvious response bias, or completion times of less than 2 min to ensure data quality and study validity.

#### Data analysis

Latent profile analysis was performed using Mplus 8.3 software. We incrementally increased the number of classes in the model and evaluated model fit using fit indices. The primary fit indices included the Akaike Information Criterion (AIC), Bayesian Information Criterion (BIC), and sample-adjusted Bayesian Information Criterion (aBIC), with lower values indicating better model fit (Qin et al., 2025). The entropy index, ranging from 0 to 1, was used to assess classification accuracy, with higher values indicating greater reliability of the latent profiles (Marotti et al., 2025). The Lo–Mendell–Rubin likelihood ratio test (LMR) and the Bootstrapped Likelihood Ratio Test (BLRT) were used to compare models, with a significant *p*-value (*p* < 0.05) indicating that the k-class model was superior to the (k-1)-class model (Marotti et al., 2025). Additionally, SPSS 26.0 software was used for statistical description, chi-square tests, and logistic regression analysis. For group comparisons of organizational silence levels among clinical nurses, post-hoc tests were conducted using the Least Significant Difference (LSD) method, with a significance level of *p* = 0.05.

#### Ethical considerations

This study adhered to the ethical principles outlined in the Declaration of Helsinki and received approval from the ethical committee of the First Affiliated Hospital of Air Force Military Medical University. All participants were informed of the research objectives, volunteered to participate, and retained the right to withdraw at any time.

## Results

### Scores of nurses’ differential atmosphere perception and organizational silence

The total score for differential atmosphere perception was 33.86 ± 9.79, and the total score for organizational silence was 56.49 ± 16.39. Details are presented in Table 2.

**Table 2:** Scores of differential atmosphere perception and organizational silence (*n* = 532).

Variable	Total score (x̅ ± s)	Item score (x̅ ± s)
Differential atmosphere perception		
Total score	33.86 ± 9.79	3.08 ± 0.89
Favoritism	9.20 ± 2.91	3.07 ± 0.97
Interdependence	18.37 ± 5.54	3.06 ± 0.92
Trusted role	6.29 ± 2.05	3.15 ± 1.02
Organizational silence		
Total score	56.49 ± 16.39	2.82 ± 0.82
Negative silence	18.05 ± 5.49	3.01 ± 0.92
Defensive silence	16.61 ± 5.55	2.77 ± 0.92
Prosocial silence	11.53 ± 3.65	2.88 ± 0.91
Indifferent silence	10.30 ± 3.77	2.58 ± 0.94

### Latent profiling and naming of nurses’ differential atmosphere perception

We gradually increased the number of fitted profiles according to the scores on the three dimensions of differential atmosphere perception. The study found that with the increase in the number of profiles, AIC, BIC, and aBIC gradually decreased, and when the number of categories was four, LMR(P) was not significant; thus, this study selected 3 profiles (Table 3). To evaluate the accuracy of the classification results and verify its reliability, we analyzed the correct classification probabilities for the 3 categories, reaching 0.961, 0.964, and 0.940, respectively.

**Table 3:** Latent profiling of nurses’ differential atmosphere perception fitting indices.

Profile	AIC	BIC	aBIC	LMR (*P*)	BLRT (*P*)	Entropy	Proportion (%)
1	8121.039	8146.596	8127.551				
2	7568.162	7610.758	7579.016	<0.001	<0.001	0.924	18.84/81.26
3	7110.811	7170.445	7126.006	0.005	<0.001	0.904	16.25/57.55/26.20
4	6996.06	7072.732	7015.596	0.056	<0.001	0.920	7.27/12.62/24.85/55.26
5	6844.21	6937.92	6868.086	0.476	<0.001	0.930	12.62/7.27/49.14/25.24/5.73

**Figure 1:**
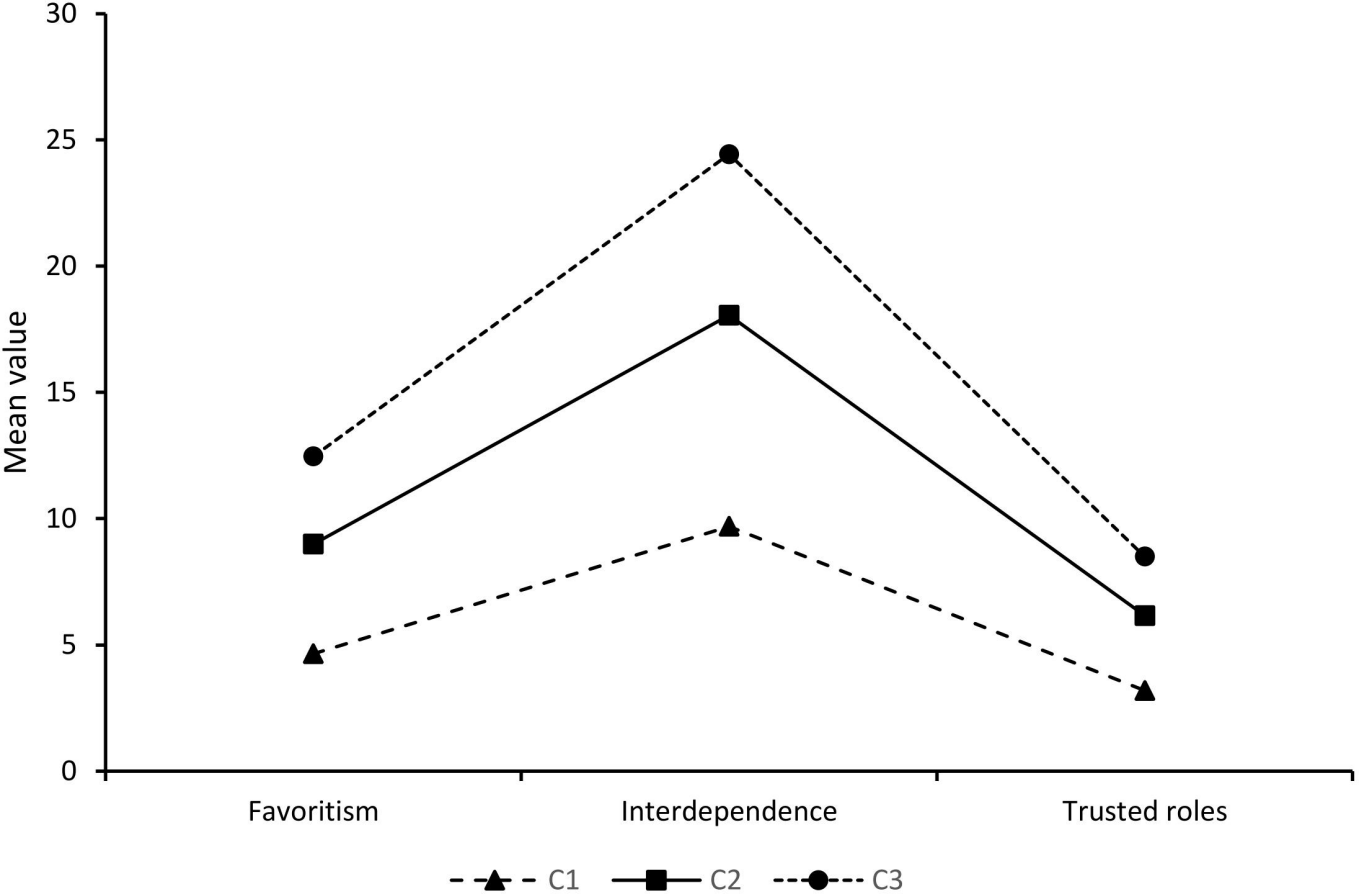
Three latent profiles of nurses’ differential atmosphere perception.

Figure 1 presents the scores for each profile in the three dimensions of differential atmosphere perception. The x-axis represents the basic characteristics of differential atmosphere perception and the y-axis represents the mean score. Profile 1 (C1) scored low on all dimensions of differential atmosphere perception, with a total of 85 clinical nurses (16.25%), leading to the categorization of this group as “insiders.” While Profile 2 (C2) scored moderately on each dimension, this category was named “Go-Betweens” and consisted of 301 people (57.55%). Scoring high on all three dimensions of differential atmosphere perception was Profile 3 (C3) with 137 or 26.20%. Due to the fact that this group of nurses perceived the most differential atmosphere, we named C3 “Outsiders.”

### Bivariate analysis of different profiles of differential atmosphere perception

One-way ANOVA revealed significant differences among the three profiles in terms of work experience (*χ*^2^ = 7.449, *p* < 0.001) and monthly income (*χ*^2^ = 6.812, *p* < 0.001). The detailed information was shown in Table 4.

**Table 4:** Bivariate analysis of different latent profiles of nurses’ differential atmosphere perception.

Variable	C1 (*n* = 85)	C2 (*n* = 301)	C3 (*n* = 137)	*χ* ^2^	*P*
Gender				3.567	0.168
Male	0 (0.0%)	11 (3.7%)	6 (4.4%)		
Female	85 (100.0%)	290 (96.3%)	131 (95.6%)		
Age				2.401	0.662
20 ~ 30	55 (64.7%)	170 (56.5%)	76 (55.5%)		
31 ~ 40	25 (29.4%)	111 (36.9%)	53 (38.7%)		
>40	5 (5.9%)	20 (6.6%)	8 (5.8%)		
Education				2.744	0.254
College degree	34 (40.0%)	93 (30.9%)	42 (30.7%)		
Bachelor’s degree or above	51 (60.0%)	208 (69.1%)	95 (69.3%)		
Title				8.642	0.071
Primary nurse	33 (38.8%)	75 (24.9%)	45 (32.8%)		
Senior nurse	38 (44.7%)	146 (48.5%)	62 (45.3%)		
Nurse-in-charge and above	14 (16.5%)	80 (26.6%)	30 (21.9%)		
Years of nursing work				7.449	<0.001
1–5 years	41 (48.2%)	116 (38.5%)	58 (42.3%)		
6–10 years	30 (35.3%)	109 (36.2%)	38 (27.7%)		
>10 years	14 (16.5%)	76 (25.2%)	41 (29.9%)		
Department				4.442	0.617
Internal medicine	46 (54.1%)	177 (58.8%)	76 (55.5%)		
Surgery	20 (23.5%)	46 (15.3%)	22 (16.1%)		
ICU/operating room/emergency department	9 (10.6%)	44 (14.6%)	20 (14.6%)		
Others	10 (11.8%)	34 (11.3%)	19 (13.9%)		
Days of night shift monthly				4.907	0.297
0	11 (12.9%)	37 (12.3%)	10 (7.3%)		
1 ~ 7	30 (35.3%)	84 (27.9%)	46 (33.6%)		
>7	44 (51.8%)	180 (59.8%)	81 (59.1%)		
Monthly income (CNY)				6.812	<0.001
<5,000	20 (23.5%)	48 (15.9%)	32 (23.4%)		
5,001 ~ 1,000	24 (28.2%)	104 (34.6%)	45 (32.8%)		
10,001 ~ 15,000	32 (37.6%)	103 (34.2%)	45 (32.8%)		
>15,000	9 (10.6%)	46 (15.3%)	15 (10.9%)		

C1: Insiders; C2: Go-betweens; C3: Outsiders; CNY: Chinese Yuan.

### Logistic regression analysis of nurses’ differential atmosphere perception profiles

Using the clinical nurses’ differential atmosphere perception categories as the dependent variable (with the “Outsiders” group as the reference group), monthly income and work experience were included as independent variables in a multivariate unordered logistic regression analysis. The coding scheme was as follows: work experience: 1–5 years = 1, 6–10 years = 2, >10 years = 3; monthly income: <5,000 CNY = 1, 5,001–10,000 CNY = 2, 10,001–15,000 CNY = 3, > 15,000 CNY = 4. The results indicated that, compared to the “Outsiders” group, clinical nurses with 1–5 years and 6–10 years of work experience were more likely to belong to the “Insiders” and “Go-betweens” groups. In contrast, clinical nurses with a monthly income of <5,000 CNY were more likely to belong to the “Outsiders” group. Details are presented in Table 5.

**Table 5:** Logistic regression analysis of nurses’ differential atmosphere perception profiles.

Dependent variable	Independent variable	β	*SE*	*P*	OR (95% CI)
C1 vs. C3	Constant	−0.747	0.441	0.090	
	Work experience (Ref: >10 years)				
	1–5 years	1.108	0.469	0.018	3.030 (1.207 ~ 7.603)
	6–10 years	0.958	0.424	0.024	2.608 (1.135 ~ 5.991)
C2 vs.C3	Constant	0.987	0.304	0.001	
	Work experience (Ref: >10 years)				
	6–10 years	0.622	0.290	0.032	1.863 (1.056 ~ 3.287)
	Monthly income (Ref: >15,000 CNY)				
	<5,000 CNY	−1.123	0.473	0.018	0.325 (0.129 ~ 0.823)

C1: Insiders; C2: Go-betweens; C3: Outsiders; ref.: reference; CNY: Chinese Yuan.

### Relationships between latent profiles of nurses’ differential atmosphere perception and organizational silence

As shown in Table 6, a differential comparison of organizational silence levels among nurses with differential atmosphere perception profiles revealed that the difference in the four dimensions of organizational silence among nurses with different profiles was statistically significant (*p* < 0.001).

**Table 6:** Differential analysis of three profiles of differential atmosphere perception and organizational silence.

Profile	Negative silence	Defensive silence	Prosocial silence	Indifferent silence	Total organizational silence
C1	12.82 ± 4.73	11.39 ± 4.55	8.21 ± 3.34	6.81 ± 2.42	39.24 ± 12.53
C2	17.67 ± 4.36	16.49 ± 4.51	11.54 ± 2.97	10.38 ± 3.11	56.09 ± 12.76
C3	22.14 ± 5.08	20.11 ± 5.59	13.55 ± 3.71	12.28 ± 4.24	68.08 ± 15.92
*Hc*	162.389	130.895	117.021	113.756	156.826
*P*	<0.001	<0.001	<0.001	<0.001	<0.001
PostHoc*	3 > 2 > 1	3 > 2 > 1	3 > 2 > 1	3 > 2 > 1	3 > 2 > 1

C1: Insiders; C2: Go-betweens; C3: Outsiders; * represents *p* < 0.05.

## Discussion

### Differential atmosphere perception and organizational silence are in the medium level

This study indicated that clinical nurses’ differential atmosphere perception was generally at a moderate level, consistent with previous research (Zhang et al., 2022). This may be attributed to the varying degrees and styles of differential treatment by clinical department leaders, manifested in poor communication, uneven workload distribution, and perceived inequitable allocation of organizational resources. Among these, the “trusted role” dimension had the highest mean item score, indicating that department managers overly trust certain subordinates. Conversely, the “interdependence” dimension had the lowest mean item score, suggesting a close emotional bond between managers and specific subordinates.

The study also revealed that nurses’ organizational silence was generally at a moderate level, with the “negative silence” dimension having the highest mean item score, consistent with the findings of Atalla et al. (2022) and Zhang et al. (2024). This may be due to nurses’ fear of damaging interpersonal relationships, apprehension about offending leaders, and a lack of confidence and security (Yağar and Dökme, 2023). Furthermore, research has shown that organizational silence not only affects patient safety but also hinders improvement and innovation in healthcare institutions (Zou et al., 2025). It is suggested that nursing managers should pay attention to the phenomenon of differential treatment and the current state of organizational silence. They should strive to create a fair, harmonious, and trusting organizational environment, encouraging clinical nurses to participate in departmental management and providing effective strategies to address these issues.

### Group heterogeneity in nurses’ differential atmosphere perception

We identified three profile of nurses’ differential atmosphere perception through latent profile analysis: Insiders (C1), Go-betweens (C2), and Outsiders (C3), accounting for 16.25, 57.55, and 26.20% of the sample, respectively. This indicated significant heterogeneity in clinical nurses’ perceptions of differential treatment, with over 80% of nurses falling into the C2 and C3 categories, highlighting the need for urgent attention to this issue. For the “Outsiders” group (C3), it is recommended that nursing managers engage in in-depth communication with clinical nurses, strengthen trust-building within departments, optimize management styles, improve compensation and performance distribution systems, protect nurses’ rights and status, and provide more development opportunities and platforms to encourage further education and career advancement. For the “Go-betweens” group (C2), managers should enhance the frequency and quality of communication and promote diversified incentive mechanisms to meet nurses’ diverse needs. For the “Insiders’ group (C1), managers should provide basic welfare guarantees and psychological support, establish feedback mechanisms and problem-solving channels, and promptly address nurses’ concerns and demands.

### Correlates of potential profiles of nurses’ differential atmosphere perception

We further explored the factors of differential atmosphere perception by the logistic regression analysis. The finding revealed that work experience and monthly income were significant factors influencing the differential atmosphere perception. Compared to the “Outsiders” group, nurses with 1–5 years and 6–10 years of work experience were more likely to belong to the “Insiders” and “Go-betweens” groups, which aligned with previous research (Zhang et al., 2021). Nurses with longer work experience tend to have a higher perception of differential treatment. This may be because, with increased work experience, clinical nurses accumulate more clinical knowledge and insights into nursing management, enabling them to better recognize disparities in power, treatment, and resource allocation within departments. Additionally, when comparing the “Go-betweens” group to the “Outsiders” group, nurses earning < 5,000 CNY per month were more likely to be in the “Outsiders” group, which was not entirely consistent with the results of previous studies (Zhang et al., 2021). Zhang et al. (2021) found no statistically significant relationship between monthly income and differential atmosphere perception. This discrepancy may be due to differences in survey timing, sample size, and statistical methods. The current study suggests that lower income levels are associated with higher perceptions of differential treatment, possibly because nurses with lower incomes face greater economic pressure, making them more sensitive to income disparities and leading to psychological imbalance, reduced motivation, and lower professional identity. Therefore, it is recommended that clinical managers ensure fair and transparent compensation systems, provide ongoing leadership training, and improve both economic and non-economic incentive mechanisms to alleviate the stress caused by power disparities.

### Latent profile differences in nurses’ organizational silence

The results showed significant differences in organizational silence levels among clinical nurses across different categories of differential atmosphere perception, with significant differences observed in all dimensions of organizational silence. According to social exchange theory, the interaction between clinical nurses and departments is essentially a reciprocal social exchange (Chen et al., 2024). When a high level of differential treatment exists, this reciprocity is severely disrupted, leading nurses to perceive organizational inequities, which can trigger negative emotions and attitudes, significantly reducing their sense of organizational identity and belonging (Lu et al., 2022). To maintain interpersonal relationships, nurses may resort to silence. Previous research has shown that the process of achieving social exchange goals often involves uncertainty and risk, which are amplified in high-differential-treatment environments, further suppressing nurses’ willingness to voice their opinions and leading to increased organizational silence (Wang et al., 2025). For department managers, addressing the negative effects of differential atmosphere is a critical challenge. Nursing managers should deepen their understanding of human resource management, optimize incentive mechanisms, and innovate human-centered management practices. At the same time, managers should focus on developing their leadership skills, transforming leadership styles, eliminating harmful leadership behaviors, and reducing differential treatment to mitigate organizational silence.

### Limitations

This study has several limitations. First, due to the cross-sectional nature of the research, it is unable to establish causal relationships between the independent and dependent variables. Second, the convenience sampling method was used, and the study was limited to clinical nurses from only three hospitals, which restricted the generalizability and external validity of the findings. Third, due to the QR-code-based distribution through institutional channels, the exact number of nurses who received the survey invitation could not be determined, precluding traditional response rate calculation. Finally, the use of self-reported questionnaires may introduce some degree of social desirability bias.

### Implications

Despite its limitations, this study has some implications for nursing administrators and researchers internationally. For nursing management, the concepts of differential climate and organizational silence have the potential to stimulate international reflection and draw attention to organizational issues, despite the fact that they are both rooted in elements and dynamics specific to Chinese society. A national survey in the United States found that more than 1 in 4 nurses said they intended to leave the profession (Suran, 2023), and surveys from 5 European countries found that almost 1 in 3 critical care nurses were considering quitting (Llaurado-Serra et al., 2025). Overwork and understaffing were rampant (Dall'Ora et al., 2025; Needleman, 2025; Suran, 2023). This is highly detrimental to the health of nurses and the efficiency of national healthcare system as a whole (Anderer, 2024; Berthelsen and Hansen, 2024). Therefore, international nursing managers need to prioritize organizational management factors, work environment factors and resource rationalization. In terms for nursing research, future research should expand the sampling scope to explore the underlying mechanisms influencing clinical nurses’ differential atmosphere perception. Additionally, qualitative interviews could be conducted to delve deeper into the factors affecting clinical nurses’ perceptions, providing a more comprehensive basis for developing targeted policies.

## Conclusion

In conclusion, nurses’ differential atmosphere perception and organizational silence were generally at moderate levels. The differential atmosphere perception was categorized into three latent profiles: “Insiders,” “Go-betweens,” and “Outsiders” groups. Work experience and monthly income were identified as influencing factors of nurses’ differential atmosphere perception, and higher levels of differential atmosphere perception (“Outsiders”) may exacerbate organizational silence. Nursing managers should create equity-based environments, improve leadership styles, move away from differential treatment and develop targeted interventions to reduce organizational silence among nurses and inspire the nursing workforce.

## Data Availability

The original contributions presented in the study are included in the article/supplementary material, further inquiries can be directed to the corresponding author.
